# Strain‐Preserving Transfer of Freestanding Oxide Membranes for Tunable Magnetic Anisotropy

**DOI:** 10.1002/advs.75845

**Published:** 2026-05-26

**Authors:** Jinfeng Zhang, Yuyue Zhao, Shouzhe Dong, Jingdi Lu, Qing Wang, Fang Xu, Ao Wang, Kunjie Dai, Yueming Huang, Zhengguo Liang, Qiming Lv, Houbing Huang, Wenbin Wu, Lingfei Wang

**Affiliations:** ^1^ Hefei National Research Center for Physical Sciences At Microscale University of Science and Technology of China Hefei China; ^2^ School of Interdisciplinary Science Beijing Institute of Technology Beijing China

**Keywords:** flexible spintronics, freestanding oxide membranes, magnetic anisotropy, strain engineering, transition‐metal oxide

## Abstract

Epitaxial strain has been widely used as an effective approach for harnessing magnetic anisotropy (MA) of complex oxide‐based heterostructures, which enables essential building blocks for advanced spintronic applications. However, extending this strain engineering of MA to flexible magnetic systems and devices is largely hindered by strain relaxation during the exfoliation and transfer procedures of freestanding oxide membranes. To address this challenge, we develop an epoxy‐assisted transfer (EAT) approach that effectively preserves the epitaxial strain state in freestanding oxide membranes. Using this approach, we impose compressive strain on the ferromagnetic freestanding La_2/3_Sr_1/3_Mn_0.9_Ru_0.1_O_3_ films and achieve robust perpendicular magnetic anisotropy (PMA) up to 4.2 × 10^5^ J/m^3^. This strain‐preserving transfer recipe can be further applied to a variety of ferromagnetic oxide membranes, such as La_2/3_Ca_1/3_MnO_3_ and SrRuO_3_, for stabilizing uniaxial in‐plane MA and even tilted PMA. Our work addresses the long‐standing trade‐off between structural flexibility and control of MA in oxide‐based heterostructures, offering versatile and innovative design strategies of flexible spintronic devices with customizable functionality.

## Graphical abstract

An epoxy‐assisted exfoliation and transfer strategy circumvents the trade‐off between structural flexibility and strain tunability in freestanding oxide membranes. Epitaxial strain is effectively preserved, enabling versatile and robust control of magnetic anisotropy without sacrificing flexibility. In freestanding La_2/3_Sr_1/3_Mn_0.9_Ru_0.1_O_3_ membranes, it yields a strong perpendicular magnetic anisotropy (4.2 × 10^5^ J/m^3^) while also enabling effective bending modulation.

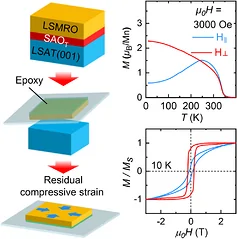

## 1 Introduction

The development of spintronic devices heavily relies on precise control of spin orientation in magnetic films, which requires tailored magnetic anisotropy (MA) along specific axes [1, 2, 3]. For instance, perpendicular magnetic anisotropy (PMA) stabilizes out‐of‐plane magnetization and thereby enables aggressive scaling of memory cells, underpinning applications in artificial intelligence and high‐performance computing [4, 5, 6, 7]. In‐plane uniaxial MA aligns the magnetization along a specific in‐plane axis and enables highly controllable magnetization switching, facilitating the design of weak‐signal detection devices such as anisotropic magnetoresistance (AMR) sensors [8, 9, 10]. Accordingly, developing suitable and effective approaches for optimizing and tailoring MA in magnetic films is one of the most essential tasks for advancing spintronic technologies.

Strain engineering has emerged as a powerful approach for tuning MA in epitaxial film systems, especially in transition‐metal oxide heterostructures, where the lattice, orbital, spin, and charge degrees of freedom are strongly coupled [11, 12, 13]. Strain‐induced structural modifications alter the crystal field environment of transition metal cations, leading to preferential occupation of specific *d*‐orbitals and considerable MA through spin‐orbit coupling (SOC) [14, 15, 16]. For example, in compressively‐strained La_2/3_Sr_1/3_MnO_3_ (LSMO) and La_2/3_Sr_1/3_Mn_0.9_Ru_0.1_O_3_ (LSMRO) films, the preferential occupation of 3*z^2^‐r^2^* orbital of Mn cations promotes a sizable PMA [15, 17]. In the anisotropic‐strained La_2/3_Ca_1/3_MnO_3_ (LCMO) thin films, the enhanced orbital polarization along the elongated axis leads to an in‐plane uniaxial MA [18, 19]. Collectively, epitaxial strain offers a direct and efficient pathway for tailoring MA in functional oxide materials.

Flexible spintronic devices, which have inherent spintronic functionalities and structural flexibility, hold transformative potential for wearable electronics, biomedical sensing, curved displays, and human‐machine interfaces [20, 21, 22]. However, conventional strain engineering of epitaxial oxide heterostructures relies on lattice mismatch and strong chemical bonding to the single‐crystal substrate surface. The millimeter‐scale thickness and high rigidity of the substrate pose a major challenge in applying functional oxide heterostructures for flexible spintronic devices. Recently developed freestanding oxide membrane exfoliating and transferring technologies are the key to addressing this limitation [23, 24, 25, 26]. Particularly, water‐assisted membrane exfoliation based on cubic‐structured Sr_3_Al_2_O_6_ (SAO_C_) sacrificial layer has emerged as a versatile and scalable approach, accelerating the integration of perovskite oxide heterostructures with advanced semiconductor technologies [23, 27, 28, 29]. While these approaches liberate oxide thin films from substrate constraints, they simultaneously remove the foundation of traditional strain engineering. First, the unavoidable lattice mismatch between the SAO_C_ sacrificial layer and the target oxide thin film typically triggers partial or full strain relaxations and even the formation of dislocations. Second, the peeling process further releases the residual strain and even causes the formation of microcracks. Accordingly, developing alternative strategies to achieve strain‐mediated control of MA in freestanding oxide membranes is highly desirable [14, 30, 31, 32].

Recently, a series of sacrificial layers capable of preserving epitaxial strain during growth has been developed [24, 33, 34]. Notably, the Sr_4_Al_2_O_7_ (SAO_T_) sacrificial layer exhibits superior flexibility under epitaxial strain, enabling coherent growth of high‐quality epitaxial heterostructures and considerably improving the crystallinity and integrity of water‐released freestanding oxide membranes [24]. In this work, based on the coherent‐strained SAO_T_ sacrificial layer, we demonstrate an epoxy‐assisted exfoliation and transfer recipe that circumvents the long‐standing trade‐off between structural flexibility and strain tunability in freestanding oxide membranes. Using this recipe, we effectively preserve the epitaxial strain state in magnetic freestanding oxide membranes after exfoliation, thereby largely maintaining the strain‐induced uniaxial MA. Using this approach, we impose compressive strain to the ferromagnetic freestanding LSMRO films and achieve robust PMA up to 4.2×10^5^ J/m^3^. This strain‐preserving transfer recipe can be further applied to a variety of ferromagnetic oxide membranes, such as LCMO and SrRuO_3_ (SRO), for stabilizing uniaxial in‐plane MA and even tilted PMA. Our work establishes a versatile and effective strategy for harnessing MA in freestanding oxide membranes, enabling innovative designs of oxide‐based spintronic devices that combine structural flexibility, low‐power consumption, ultrafast, and ultrasensitive.

## 2 Results and Discussion

**FIGURE 1:**
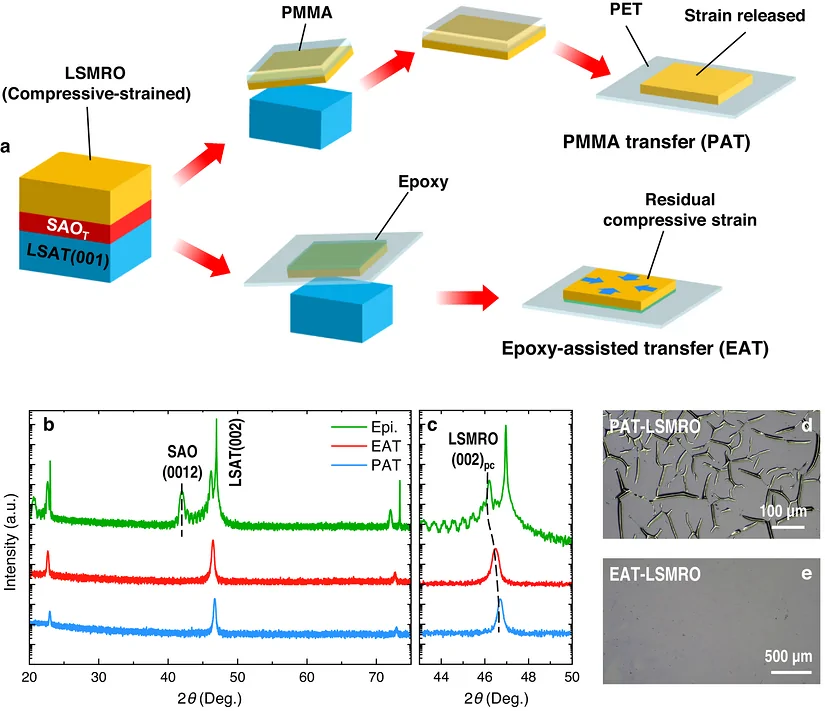
Preparation and structural characterizations of freestanding LSMRO membranes. (a) Schematic illustrations of two transfer recipes for preparing the freestanding LSMRO membranes. (b,c) XRD 2*θ‐ω* linear scans of a 30 nm thick LSMRO/SAO_T_/LSAT(001) epitaxial thin film (denoted as Epi.) and the corresponding freestanding membranes prepared by EAT and PAT methods. (d,e) Optical microscopic images measured from the PAT‐ and EAT‐prepared LSMRO membranes.

Figure 1a schematically illustrates two experimental methods for constructing freestanding single‐crystalline films based on the SAO_T_ sacrificial layers. The key distinction between these methods lies in the supporting layer, which essentially affects the strain state and physical properties of the transferred oxide membranes. For the traditional polymethyl methacrylate (PMMA)‐assisted transfer (PAT) recipe, a PMMA supporting layer is spin‐coated onto the oxide membranes, forming physical adsorption and providing mechanical stability during water‐release. Then the exfoliated oxide membrane can be directly transferred to various flexible substrates [e.g., polyethylene terephthalate (PET) sheets]. Finally, the PMMA support layer is removed by acetone. During these water‐release and transfer procedures, the epitaxial strain imposed by the substrate is expected to be fully released. We also developed an epoxy‐assisted transfer (EAT) method: as detailed in the Experimental Section of the Supporting Information, a two‐component epoxy resin is spin‐coated onto the cleaned oxide/SAO_T_ epitaxial film surface and laminated with a PET sheet before water‐release. After curing, the epoxy layer provides a much stronger mechanical constraint than PMMA, and its thickness is approximately 3 µm (Figure S1). The PET/epoxy/oxide/SAO_T_/substrate stack is then immersed in deionized water to dissolve the sacrificial layer, after which the PET/epoxy/oxide membrane laminate is directly retrieved after substrate detachment. In this case, the cured epoxy resin forms strong secondary bonds with the oxide membrane surface due to its abundant polar groups (such as epoxy and hydroxyl groups), and the resulting continuous support throughout the release and transfer process may help preserve the residual epitaxial strain in the water‐released oxide membranes.

We grow 30 nm‐thick LSMRO film epitaxially on a 20 nm SAO_T_‐buffered (001)‐oriented (LaAlO_3_)_0.3_‐(SrAl_0.5_Ta_0.5_O_3_)_0.7_ [LSAT(001)] substrate via pulsed laser deposition (See Experimental Section in the Supporting Information for details), and then applied the two aforementioned transfer recipes to prepare the freestanding LSMRO membranes. In pseudocubic notation, the in‐plane lattice constant (*a*_pc_, where “pc” stands for pseudocubic lattice coordinates) of bulk LSMRO is 3.884 Å, 0.414% larger than that of the LSAT substrate (*a*_pc_ = 3.868 Å). Therefore, the LSMRO film epitaxially grown on SAO_T_/LSAT(001) substrate is expected to be biaxially and compressively strained. As shown in Figure 1b,c, the XRD 2*θ‐ω* scan of the epitaxial LSMRO/SAO_T_/LSAT(001) films shows strong LSMRO(00*l*) peaks and well‐defined Laue fringes, confirming high epitaxial quality with the presence of the SAO_T_ buffer layer. The out‐of‐plane lattice constant of LSMRO in pseudo‐cubic notation (*c*_pc_) is 3.926 Å, much larger than the bulk value, which can be attributed to the compressive strain imposed by the LSAT(001) substrate. For the freestanding LSMRO membranes prepared by the EAT method, the LSMRO(00*l*) diffraction peaks shift slightly toward higher Bragg angles compared to those of the epitaxial film. But the *c*_pc_ value (3.905 Å) is still higher than the bulk value, signifying the existence of residual compressive strain. For the freestanding LSMRO membranes prepared by the PAT method, the LSMRO(00*l*) diffraction peaks show a more significant shift toward higher Bragg angles. And the *c*_pc_ = 3.884 Å is identical to the bulk value, strongly suggesting a full relaxation of compressive strain after water‐release and transfer [35]. We further evaluate the integrity and surface morphology of freestanding LSMRO membranes by optical microscopic images (Figure 1d,e). As shown in Figure 1d, for the freestanding LSMRO membranes prepared by the PAT recipe, the optical microscopic image barely shows any signature of microcracks but displays high‐density wrinkles, probably formed due to strain relaxation during the water‐release process. In contrast, for the membrane prepared by the EAT method, the optical image does not show any sign of cracks or wrinkles, displaying a much smoother and cleaner surface compared to the PAT‐prepared sample (Figure 1e).

**FIGURE 2:**
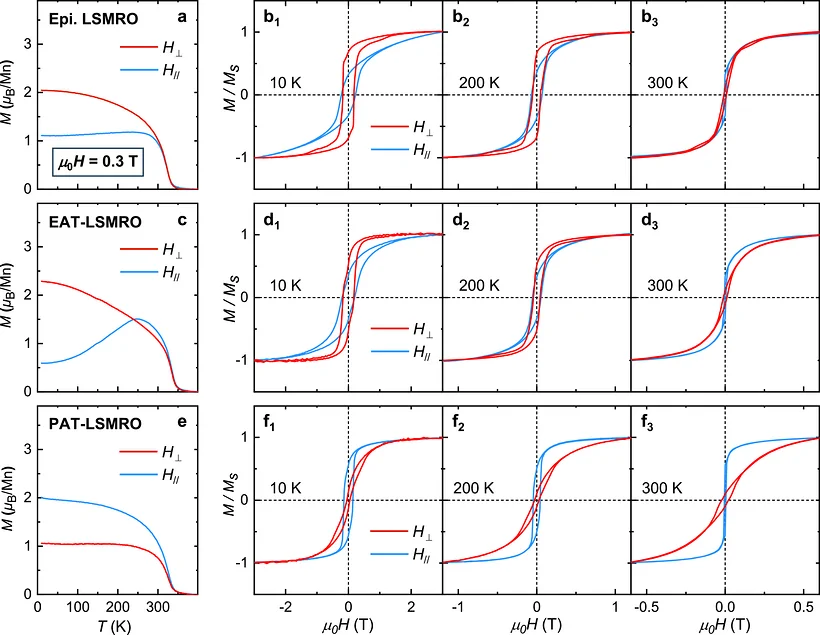
Magnetic characterizations of the LSMRO epitaxial film and freestanding membranes. (a,b) *M‐T* curves (a) and *M‐H* hysteresis loops (b) measured from the 30 nm‐thick epitaxial LSMRO/SAO_T_/LSAT(001) films. For the *M‐T* measurements, a static magnetic field 𝜇_0_𝐻 = 3000 Oe is applied either along the in‐plane [010]_pc_ axis (𝐻_∥_, blue) or along the film normal (𝐻_⊥_, red). The *M‐H* loops are measured at 10 K, 200 K, and 300 K. (c,d) *M‐T* and *M‐H* curves measured from the EAT‐prepared freestanding LSMRO membranes. (e,f) *M‐T* and *M‐H* curves measured from the PAT‐prepared freestanding LSMRO membranes.

We then investigate the impact of different transfer methods on the magnetic properties of freestanding LSMRO membranes. To comprehensively evaluate the MA of LSMRO epitaxial thin films and freestanding membranes, as shown in Figure 2 and Figure S2, we conducted detailed magnetic characterization with an external magnetic field (*H*) applied along either the in‐plane LSMRO[010]_pc_ axis (*H*_∥_) and out‐of‐plane LSMRO[001]_pc_ axis (*H*_⊥_). Temperature‐dependent magnetization (*M‐T*) curves of epitaxial LSMRO/SAO_T_/LSAT(001) film (Figure 2a) show that the *M* measured with 𝐻_⊥_ (denoted as *M*_⊥_) is much larger than that measured with *M*_∥_, evidencing a considerable PMA below the Curie temperature (*T*_C_). The evolution of MA with *T* can be further revealed via *H*‐dependent magnetization (*M‐H*) hysteresis loops. At 10 K, the *M*_⊥_ increases and saturates rapidly with *H*_⊥_, while the increase of *M*_∥_ with *H*_∥_ becomes rather slanted (Figure 2b_1_), indicating a strong PMA. As *T* increases to 200 and 300 K, the *M‐H* curves measured with *H*_⊥_ and *H*_∥_ tend to be much more isotropic (Figure 2b_2_,b_3_), indicating a decay of PMA upon warming [17]. For the freestanding LSMRO membranes prepared by the EAT method, the *M‐T* curves show that the *M*_∥_ drops sharply when cooling below 250 K and becomes markedly smaller than the *M*_⊥_, suggesting an even more pronounced enhancement of PMA upon cooling (Figure 2c). The *M‐H* curves measured at 10 K (Figure 2d_1_) also signify a strong PMA. As *T* increases from 10 K to 200 and 300 K, the *M*‐*H*_⊥_ curves gradually evolve from a rectangular hysteresis loop to a narrow and slanted loop (Figure 2d_2_,d_3_). According to the Stoner‐Wohlfarth model [36], this *M‐H* evolution reflects a progressive reorientation of the magnetic easy axis from film normal to in‐plane [13, 37]. For the freestanding LSMRO membranes prepared by the PAT method, in sharp contrast, both the *M‐T* and *M‐H* curves demonstrate an in‐plane MA over the entire *T* range (Figure 2e,f). And the nearly identical *M‐T* and *M‐H* curves measured with variable in‐plane *H* orientation further confirmed a demagnetization‐dominated magnetic easy plane behavior (Figure S3). These magnetic characterization results underscore the decisive role of the transfer recipe in controlling the MA of the LSMRO freestanding membrane.

**FIGURE 3:**
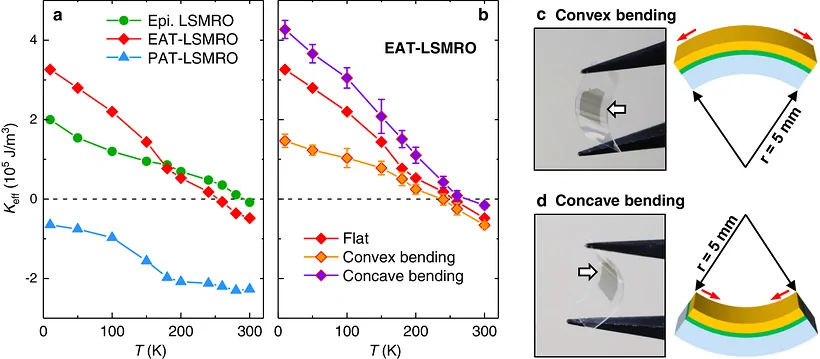
Evolutions of magnetic anisotropy of EAT‐prepared freestanding LSMRO membranes with *T* and mechanical bending. (a) *T*‐dependence of the effective magnetic anisotropy energy (*K*_eff_) for epitaxial LSMRO films as well as the EAT‐ and PAT‐prepared freestanding LSMRO membranes. (b) *K*_eff_ ‐*T* curves for EAT‐prepared LSMRO membranes in the flat, convex bending, and concave bending configurations. The error bars represent the standard deviations of *K*_eff_ over multiple bending cycles. (c,d) Optical photographs (left panels) and schematic illustrations (right panels) of the (c) convex and (d) concave bending configurations used for introducing tensile/compressive strain states in the freestanding LSMRO membranes transferred onto PET.

To further reveal the evolutions of MA in freestanding LSMRO membranes, we extract the effective magnetic anisotropic energy (*K*_eff_) from the *M‐H* loops shown in Figure 2 and Figure S2, using the following equation:

(1) $$K_{\mathrm{eff}}=\frac{M_{s}\cdot H_{a}}{2}$$

where *M_s_* is the magnetization at the saturation field *H*_s_. And the *H*_a_ is the anisotropic field, calculated by subtracting the *H*_s_ measured in the 𝐻_⊥_ case from the ones measured in 𝐻_∥_ case. The positive and negative signs of *K*_eff_ represent PMA and in‐plane MA, respectively. As summarized in Figure 3a, the *K*_eff_ of LSMRO/SAO_T_/LSAT(001) epitaxial films maintains positive over the entire *T* range from 10 to 300 K, consistent with strong PMA observed from the *M‐T* and *M‐H* curves. And the *K*_eff_ value increases progressively upon cooling, probably due to the increased *M* and orbital polarization [38]. In contrast, the *K*_eff_ of PAT prepared membranes consistently remains negative over the entire *T* range, confirming a robust in‐plane MA. Notably, for the EAT‐LSMRO membranes, the *K*_eff_ shows a sign reversal at ∼250 K, suggesting a reorientation of magnetic easy axis from in‐plane to out‐of‐plane upon cooling: at *T* > 250 K, the small and negative *K*_eff_ suggests a weak in‐plane MA; upon cooling, the *K*_eff_ increases fast and even exceeds the value of the epitaxial films at *T* < 170 K. Considering that the thermal expansion coefficients of epoxy and PET are much larger than that of LSMRO, we suggest that the epoxy/PET laminate can further impose thermal strain to the LSMRO membrane upon cooling, which results in more pronounced PMA at *T* < 170 K (Figure S4).

To verify the applicability of EAT‐LSMRO membranes in flexible spintronic devices, we further characterized the evolution of their magnetic properties upon mechanical bending. Specifically, we bent the EAT‐LSMRO membrane into convex and concave configurations, both with a fixed curvature radius of 5 mm (Figure 3c,d). Then we measured the *M‐T* and *M‐H* curves with *H*_⊥_ and *H*_∥_ for extracting the *K*_eff_ values (Figures S5 and S6). Considering the positive Poisson's ratio and positive magnetoelastic coefficient of LSMRO [17], convex (concave) bending imposes homogeneous in‐plane tensile (compressive) strain to the EAT‐LSMRO membrane (Figure S7), thus weakening (enhancing) the PMA. Consistently, as summarized in Figure 3b, compared to the flat case, the convex bending configuration produces a pronounced reduction in *K*_eff_, whereas concave bending yields an obvious increase in *K*_eff_. These bending‐induced modulations in *K*_eff_ prevail over the entire *T* range from 300 to 10 K. These results demonstrate robust, reversible control of PMA in transferred LSMRO membranes under mechanical bending, suggesting that the EAT‐LSMRO membrane is a suitable building block for flexible spintronic devices.

**FIGURE 4:**
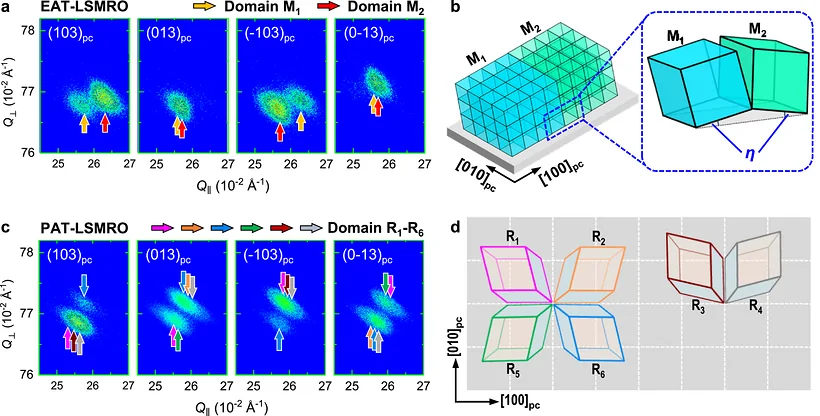
RSM‐revealed structural domain configurations of freestanding LSMRO membranes. (a,c) Off‐specular RSMs measured from the (a) EAT‐prepared and (c) PAT‐prepared LSMRO membranes around the (103)_pc_, (013)_pc_, (−103)_pc_, and (0–13)_pc_ diffractions. (b,d) Schematic illustrations of the structural domain configurations in (b) EAT‐ and (d) PAT‐prepared LSMRO membranes. In (a), the diffraction spots corresponding to the M_1_‐ and M_2_‐domains are marked by the orange and red arrows, respectively. In (c), the diffraction spots corresponding to the rhombohedral R_1_‐R_6_ domains are marked by the purple, orange, brown, grey, green, and blue arrows.

To elucidate the structural origins of the highly‐tunable PMA, we employ off‐specular XRD reciprocal space mapping (RSM) characterizations on both LSMRO/SAO_T_/LSAT(001) epitaxial film and freestanding LSMRO membranes. For the RSMs acquired from the epitaxial LSMRO/SAO_T_/LSAT(001) films (Figure S8), the diffraction spots of LSMRO{103}_pc_, SAO_T_(2218), and LSAT(103) share the same in‐plane reciprocal vector (*Q*_∥_), corroborating the coherent and compressive strain state. And the unequal out‐of‐plane reciprocal vector (*Q*_⊥_) signifies a monoclinic lattice of the LSMRO epitaxial thin film. Moreover, two satellite diffractions appear beside the LSMRO(103)_pc_ and (−103)_pc_ diffractions, while the satellites disappear around the LSMRO(0±13)_pc_ reflections. These RSM features further indicate the existence of two energetically equivalent tilting distortions and a periodic twin domain modulation along [100]_pc_ direction (Figure S8) [39]. The orientation of this uniaxial twin domain modulation is determined by the step‐terrace morphology of the LSAT(001) substrate. In particular, the LSAT(001) substrate we used has a miscut angle of ∼0.1° along [100]_pc_ axis, leading to a uniform step‐terrace structure along [010]_pc_ (Figure S8c). To minimize the elastic energy cost, the periodic domain stripes preferentially align parallel to these step edges, thereby selecting the corresponding twin domain modulation along [100]_pc_ axis. Due to this uniaxial twin modulation, the LSMRO epitaxial film is coherently and compressively strained along [010]_pc_ but partially strain relaxed along [100]_pc_, resulting in an in‐plane anisotropic strain state. For the RSMs of EAT‐prepared freestanding LSMRO membranes (Figure 4a), the *Q*_⊥_ of LSMRO(013)_pc_ is higher than that of LSMRO(0‐13)_pc_, confirming the persistence of monoclinic distortion after water‐release. In contrast, both the LSMRO(103)_pc_ and (‐103)_pc_ diffractions split into two broad spots, distinct from the satellite peaks observed in the epitaxial counterpart. According to previous literature, this diffraction spot splitting could be attributed to the formation of monoclinic twinned domains (denoted as M_1_ and M_2_) [40, 41]. As schematically illustrated in Figure 4b, the twin domains share the same monoclinic structure as the epitaxial film, while the LSMRO(001)_pc_ planes of M_1_ and M_2_ domains tend to incline toward opposite directions. The opposite tilting distortions for M_1_ and M_2_ domains result in unequal *Q*_∥_ values along [100]_pc_ axis, thus leading to the lateral splitting of LSMRO(103)_pc_ and (‐103)_pc_ diffractions [42, 43]. Similar to the structural modulations in the epitaxial film case, this twin domain formation in the EAT‐LSMRO membrane largely preserves the compressive strain along [010]_pc_ but causes slight strain relaxation along [100]_pc_. For the RSM of PAT‐LSMRO membranes, all of the {103}_pc_ diffractions split into two diffused spots (Figure 4c), implying the formation of bulk‐like rhombohedral domains. And the obvious increases in the *Q*_⊥_ values of these diffraction spots further suggest a full strain relaxation in the PAT‐LSMRO membranes. As schematically depicted in Figure 4d, the RSM diffraction spots correspond to six distinct rhombohedral domains, denoted as R_1_ to R_6_ [44]. The four equivalent rhombohedral domains (R_1_‐R_4_) originate from the two monoclinic precursor twin domains in the epitaxial LSMRO film and remain compatible with the initial monoclinic tilting distortion (Figure S9). In contrast, the formation of R_5_ and R_6_ domains is associated with strain relaxation‐induced lattice flipping of domains R_1_ and R_3_ around the [100]_pc_ axis.

Having established the strain state and domain structures of the freestanding LSMRO membranes, we now try to understand the impact of strain on their magnetic properties. The MA in LSMRO films is dominated by strain‐induced single‐ion anisotropy in Ru ions and interactions between *t*_2g_ orbitals of Ru 4d and Mn 3d cations [17, 39]. We thereby suggest that the distinct MA in PAT‐ and EAT‐prepared freestanding LSMRO membranes should originate from the distinct strain states. Structural analyses reveal that the EAT‐prepared LSMRO membrane preserves a monoclinic twin domain configuration with residual compressive strain. Since the Ru‐doping can effectively enhance the SOC and the magneto‐elastic coupling strength [17, 35], the residual compressive strain in the EAT‐prepared LSMRO membranes gives rise to a sizable PMA. In contrast, structural analyses on the PAT‐LSMRO membrane reveal the formation of bulk‐like rhombohedral multidomain configuration with nearly full strain relaxation. Consequently, the demagnetization effect prevails in the PAT‐prepared LSMRO membranes and thus leads to an in‐plane MA.

**FIGURE 5:**
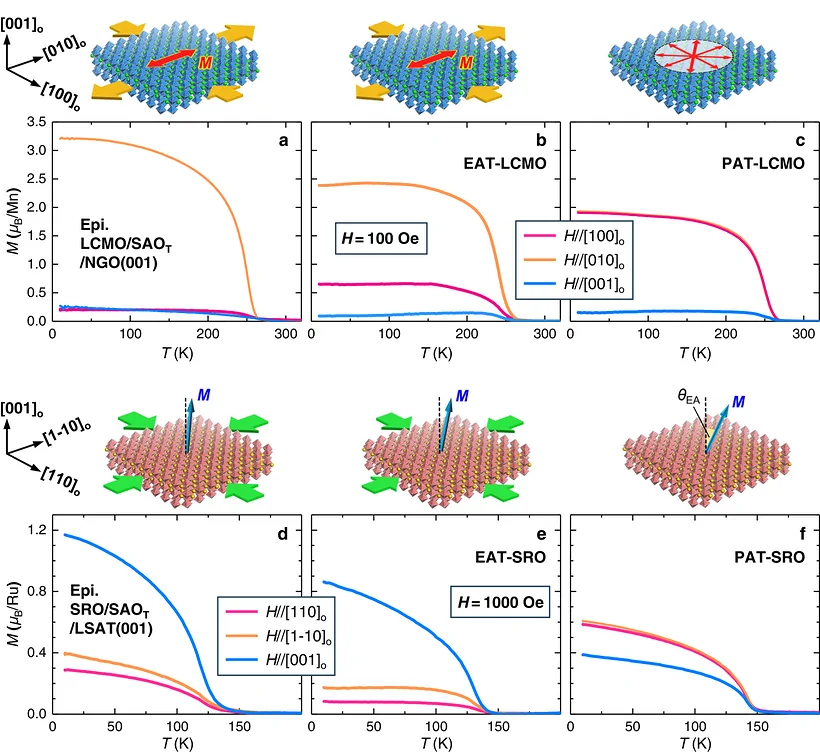
Characterizations on the MA of LCMO and SRO epitaxial films/freestanding membranes. (a‐c) *M‐T* curves of (a) LCMO/SAO_T_/NGO(001) epitaxial thin films, (b) EAT‐prepared LCMO membranes, and (c) PAT‐prepared LCMO membranes. During the measurements, we apply a magnetic field *H* = 100 Oe along three orthogonal axes: LCMO[100]_o_, LCMO[010]_o_, and LCMO[001]_o_. (d‐f) *M‐T* curves of (d) SRO/SAO_T_/LSAT(001) epitaxial thin films, (e) EAT‐SRO membranes, and (f) PAT‐SRO membranes. During the measurements, we apply a magnetic field *H* = 1000 Oe along three orthogonal axes: SRO[1‐10]_o_, SRO[110]_o_, and SRO[001]_o_, which corresponds to the LSAT[100]_pc_, LSAT[010]_pc_, LSAT[001]_pc_, respectively. The top insets of (a‐f) are schematic illustrations of the strain states and magnetization vector aligned along the magnetic easy axes.

The above results have confirmed that PMA of freestanding LSMRO membranes is dominated by EAT‐enabled residual compressive strain. Following this scenario, combining the strain‐coherent SAO_T_ sacrificial layer and EAT approach could stabilize diverse strain states and MA configurations in other freestanding magnetic oxide membranes. To test this speculation, we extended the EAT‐based strain engineering to freestanding SRO and LCMO membranes. As summarized in Table S1, the 30 nm‐thick LCMO film is epitaxially grown on SAO_T_‐buffered NGO(001) substrate, which can impose an anisotropic strain state: ‐0.71% along [100] axis and +0.85% along [010] axis. And the 35 nm‐thick SRO film is epitaxially grown on SAO_T_‐buffered LSAT(001) substrate, which can impose a bi‐axial strain of −1.58%. The XRD characterizations on the freestanding LCMO and SRO membranes confirm a consistent strain modulation as the LSMRO membranes: the PAT recipe results in a full strain relaxation, while the EAT recipe can partially preserve the epitaxial strain state (Figure S10). The distinct strain state also leads to significant modulations of the MA of LCMO and SRO. For the epitaxial LCMO/SAO_T_/NGO(001) film, the *M‐T* and *M‐H* curves measured with *H* applied along three orthogonal axes ([100]_o_, [010]_o_, [001]_o_) demonstrate a robust in‐plane uniaxial magnetic anisotropy with the magnetic easy axis along LCMO [010]_o_ axis (Figure 5a; Figure S11), originating from the NGO(001)‐imposed anisotropic strain. For the EAT‐prepared freestanding LCMO membranes, the largely preserved anisotropic strain also stabilizes a strong in‐plane uniaxial MA (Figure 5b), while the slight increment of *M* along [100]_o_ axis implies a partial strain relaxation, similar to the LSMRO case. In contrast, for the PAT‐prepared freestanding LCMO membranes, the complete strain relaxation demagnetization effect results in easy‐plane anisotropy. (Figure 5c). For the SRO films, the strong magneto‐elastic coupling results in an even more effective modulation of MA by membrane transfer recipes (Figure 5d–f). For the epitaxial SRO/SAO_T_/LSAT(001) film, the cooperation of biaxial compressive strain and orthorhombic structure of SRO results in slanted PMA: the magnetic easy axis tilted from the film normal by *θ*_EA_ = 6.6° (Figure 5d; Figure S12a). For the EAT‐SRO membrane, the biaxial compressive strain state is largely preserved, leading to a similar slanted PMA with a comparable *θ*_EA_ = 7.8° (Figure 5e; Figure S12b). Nevertheless, for the PAT‐SRO membranes, the strain‐relaxation results in a significant increase of *θ*_EA_ to 64.9° (Figure 5f; Figure S12c), which is dominated by the intrinsic magnetocrystalline MA of SRO [45].

## 3 Conclusions

In summary, we demonstrated the EAT method as a promising experimental strategy for preserving epitaxial strain state in freestanding oxide membranes. When applied to ferromagnetic oxide membranes, this approach enables efficient strain‐engineering control of MA, effectively resolving the long‐standing trade‐off between structural flexibility and controllable magnetism. The combination of inherent structural flexibility and highly tunable ferromagnetism makes the EAT‐prepared freestanding oxide membranes an attractive building block of flexible spintronic devices. Compared with widely‐used ferromagnetic metal layers (e.g., cobalt‐ or permoalloy‐based layers), the EAT‐prepared ferromagnetic membranes exhibit higher spin‐polarization, stronger MA, and much better environment and thermal stabilities, thereby offering the potential for boosting the device performance and reliability [46, 47, 48]. Moreover, the EAT strategy can be generalized to other functional oxide membranes, opening viable pathways for effective control of ferroic orderings in oxide‐based flexible electronic devices.

## Conflicts of Interest

The authors declare no conflict of interest.

## Supporting information




**Supporting File**: advs75845‐sup‐0001‐SuppMat.pdf.

## Data Availability

The data that support the findings of this study are available from the corresponding author upon reasonable request.
