# Supplementary material for: Strain‐Preserving Transfer of Freestanding Oxide Membranes for Tunable Magnetic Anisotropy
Source: Adv Sci (Weinh). 2026 May 26;13(47):e75845. doi: 10.1002/advs.75845 (PMC13335864; doi:10.1002/advs.75845)
Supplement: Supplementary file 1 [file ADVS-13-e75845-s001.pdf]

Supporting Information for

Strain-Preserving Transfer of Freestanding Oxide Membranes for Tunable Magnetic Anisotropy

*Jinfeng Zhang[1], Yuyue Zhao[1], Shouzhe Dong[2], Jingdi Lu[1], Qing Wang[1], Fang Xu[1], Ao Wang[1], Kunjie Dai[1], Yueming Huang[1], Zhengguo Liang[1], Qiming Lv[1], Houbing Huang[2], Wenbin Wu[1] and Lingfei Wang[1]**

1. Hefei National Research Center for Physical Sciences at Microscale, University of Science and Technology of China, Hefei 230026, China.
2. School of Interdisciplinary Science, Beijing Institute of Technology, Beijing, 100081, China

E-mail: wanglf@ustc.edu.cn

Experimental Section

Epitaxial film growth

ABO_3/SAO_T heterostructures were grown by a pulsed laser deposition (PLD) system equipped with a 248 nm KrF excimer laser. The SAO_T layer was grown at a substrate temperature of 650 ℃ and oxygen pressure of 5 Pa, using 1.0 J/cm^2 laser fluence. The oxides (LSMRO, LCMO, and SRO) were subsequently deposited at their optimal conditions according to previous publications. Before cooling down to room temperature at a rate of 20 °C/min, these films were in situ annealed for 10 min.

Freestanding oxide membrane preparation

i) PMMA-assisted transfer (PAT) method. The LSMRO/SAO_T/LSAT(001) epitaxial film was capped with polymethyl methacrylate (PMMA) by spin coating at 3000 rpm for 30 s. The PMMA/LSMRO/SAO_T/LSAT(001) stack was immersed in deionized water to remove the SAO_T layer, while the LSMRO membrane remained attached to the LSAT(001) substrate due to the van der Waals interactions. The PMMA/LSMRO/LSAT(001) stack was then dipped in water again. The freestanding PMMA/LSMRO sheets floated on the water surface, while the substrate sank into the water. The freestanding sheets were then picked up by polyethylene terephthalate (PET) films, and the upper PMMA protective layer was removed using acetone. Analogous procedures were used for LCMO and SRO membranes.

ii) Epoxy-assisted transfer (EAT) method. The epoxy resin used in this work is EPO-TEK 353ND, consisting of resin (Part A) and hardener (Part B). Before use, the two components were mixed at a weight ratio of 10:1 with care to minimize air entrapment, followed by degassing. The mixed epoxy was then preheated to 60 °C and spin-coated onto the cleaned LSMRO epitaxial thin film surface at 3000 rpm for 30 s. After spin coating, residual epoxy accumulated near the sample edges was gently removed, and a flexible polyethylene terephthalate (PET) sheet was immediately attached to the epoxy-coated surface to provide mechanical support. The assembled PET/epoxy/LSMRO/SAO_T/LSAT(001) stack was subsequently baked at 90 °C for 6 h to ensure complete curing of the epoxy layer, resulting in an epoxy layer approximately 3 μm. The fully cured PET/epoxy/LSMRO/SAO_T/LSAT(001) assembly was then immersed in room-temperature deionized water to dissolve the SAO_T sacrificial layer. After the full dissolution of SAO_T layer and the detachment of the rigid LSAT(001) substrate, the PET/epoxy/LSMRO stack was directly retrieved from the water. Analogous procedures were used for LCMO and SRO membranes.

Structural and magnetic characterizations

The epitaxial quality of the oxide films was analyzed by a high-resolution X-ray diffractometer (PANalytical Empyrean X-ray diffractometer, Cu Kα1 radiation) with both the 2θ-ω linear scan and off-specular reciprocal space mapping (RSM) mode. The thicknesses of oxide thin films were determined by fitting Laue fringes. Temperature and magnetic field-dependent magnetization (*M*-

T, *M-H*) curves were characterized using a vibrating sample magnetometer (VSM-SQUID, Quantum Design).

For the bending test, the samples were fixed on the non-magnetic mold with a concave or convex shape (radius = 5 mm). As illustrated in Figure S5, there are two distinct field geometries for magnetic characterizations during the bending tests. For the case of applying magnetic field in-plane the bending axis of the sample was aligned parallel to the magnetic field. For the case of applying magnetic field out-of-plane, the bending axis of the sample was perpendicular to the magnetic field.

Finite-element simulations

Finite-element simulations were performed using COMSOL Multiphysics to model the mechanical response and thermal strain distribution. The model consists of a three-layer heterostructure comprising a LSMRO functional film with a thickness of 30 nm, epoxy adhesive layer of 3 μm, and PET flexible substrate of 50 μm.

To account for mismatch-induced strain arising from differences in the coefficients of thermal expansion among the constituent materials, the total strain ε_{tot} was decomposed into elastic strain ε_{el} and thermal strain ε_{th}. The thermal strain associated with temperature variation follows the constitutive relation:

$$\varepsilon_{th} = \alpha(T - T_{ref})$$

Where α is the coefficient of thermal expansion of the respective material, T is the operating temperature, and T_{ref} is the reference (strain-free) temperature, taken as the initial ambient temperature. All materials were modeled as linear elastic and isotropic, with mechanical behavior defined by Young's modulus (E), Poisson's ratio (μ), and α. For the boundary conditions, a fixed constraint was applied at a designated reference point to suppress rigid-body motion, while all other boundaries were left free to deform. The domain was discretized using a fine mapped mesh to accurately capture the stress at the interfaces.

To obtain a bent configuration with a radius of curvature of 5 mm, a fitting strategy was employed. Specifically, a global equation incorporating an integral operator was defined to minimize the deviation between the deformed geometry and the target circular arc (R = 5 mm). The displacement field was solved iteratively under geometric nonlinearity, and the resulting strain distribution in the bent film was extracted from the solid mechanics module.

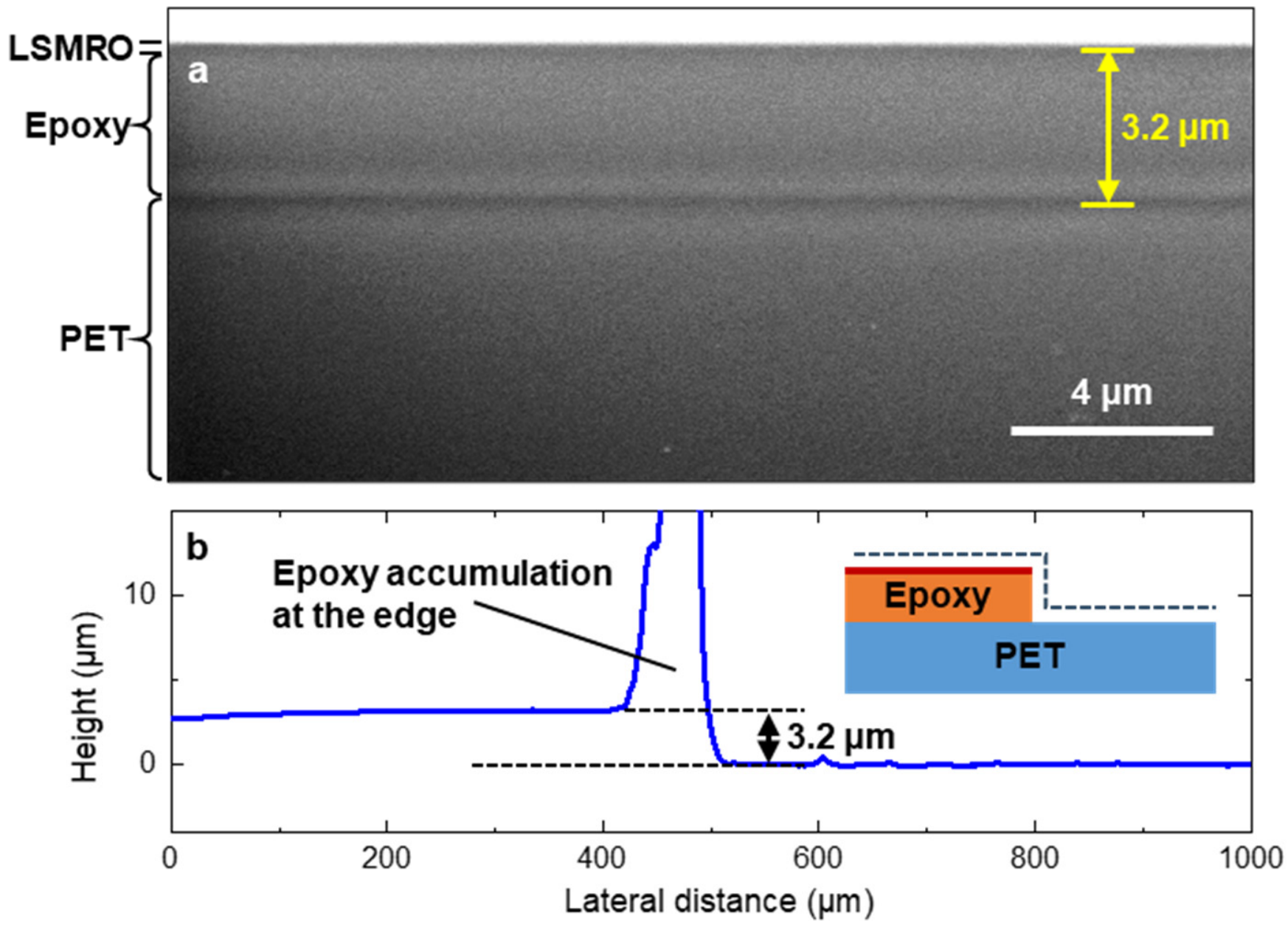

Figure S1. Thickness characterization of the epoxy layer used in the EAT process. (**a**) Cross-sectional SEM image of a FIB-prepared LSMRO/epoxy/PET specimen, revealing an epoxy thickness of approximately 3.2 µm. (**b**) Height profile measured across the boundary between the LSMRO/epoxy/PET region and the bare PET region. The step height further corroborates the presence of an epoxy layer with a thickness of approximately 3.2 µm. The high peak feature arises from the accumulation of liquid epoxy resin near the edge during PET lamination.

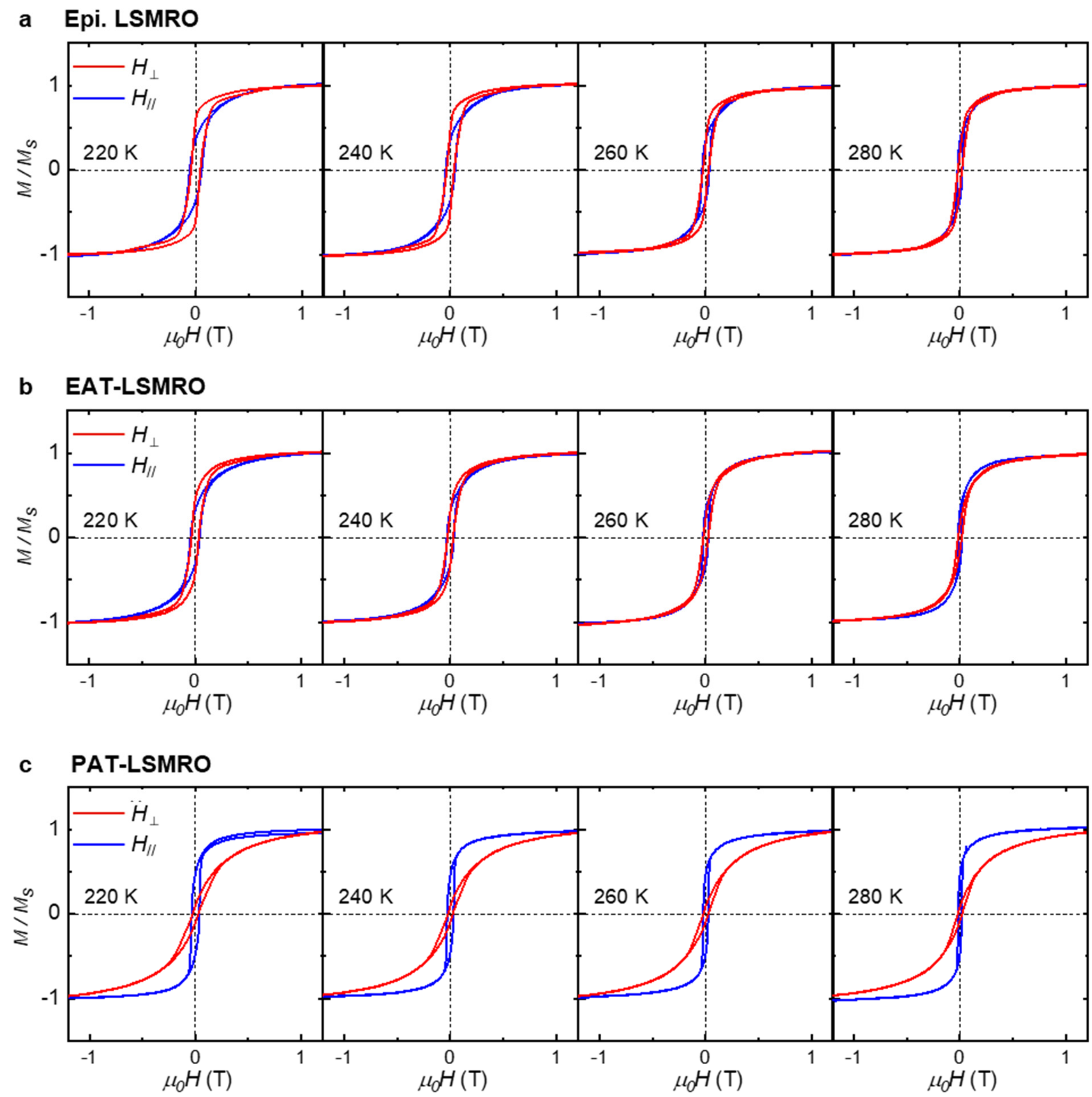

Figure S2. (**a-c**) *M-H* hysteresis loops measured at 220 K, 240 K, 260 K, 280 K from the (a) LSMRO/SAO_T/LSAT(001) epitaxial films, (b) EAT- and (c) PAT-prepared LSMRO freestanding membranes.

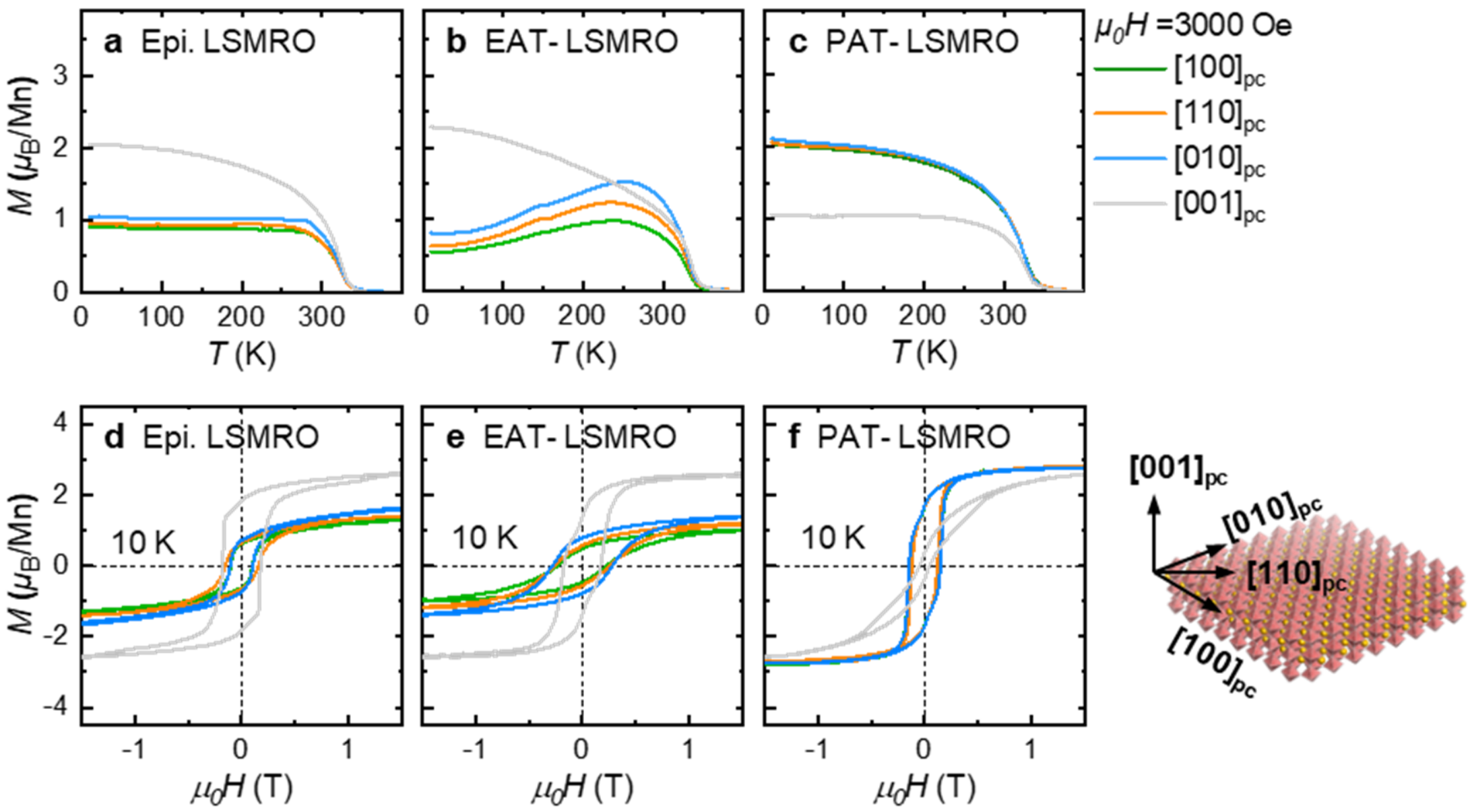

Figure S3. Magnetic characterizations of the LSMRO epitaxial film and freestanding membranes. (**a,d**) *M-T* curves (a) and *M-H* hysteresis loops (d) measured from the epitaxial LSMRO film along the in-plane $[100]_{pc}$, $[110]_{pc}$, and $[010]_{pc}$ axes, as well as the out-of-plane $[001]_{pc}$ axes. The *M-T* curves were acquired under a magnetic field of $\mu_0 H$ = 3000 Oe and the *M-H* loops were measured at 10 K. (**b,e**) *M-T* and *M-H* curves measured from the EAT-LSMRO membranes. (**c,f**) *M-T* and *M-H* curves measured from the PAT- LSMRO membranes. The lower right inset is a schematic diagram of the four axes in magnetic measurement.

The epitaxial LSMRO film exhibits a relatively weak but clearly discernible in-plane MA. In both the *M-T* and *M-H* curves, the *M* value measured along $[010]_{pc}$ ($M_{[010]pc}$) is the highest, the $M_{[100]pc}$ is the lowest, and the $M_{[110]}$ lies in between. This behavior is consistent with the in-plane anisotropy reported in related manganite systems, which often arises from the $a^+a^-c^-$-type oxygen octahedral rotation pattern and twin-domain modulation induced anisotropic strain state[1]. The EAT-prepared freestanding LSMRO membrane exhibits a similar in-plane MA as the epitaxial counterparts, namely, $M_{[010]pc} > M_{[110]pc} > M_{[100]pc}$. Compared with the epitaxial LSMRO film, the difference among the three in-plane directions becomes slightly larger, especially at the low temperature range. This behavior to the largely preserved strain along $[010]_{pc}$ axis and the disruption of the periodic structural modulation along $[100]_{pc}$, which further enhances the strain anisotropy and thus the in-plane MA.

At the same time, we would like to emphasize that, for both the epitaxial LSMRO film and the EAT-prepared LSMRO membrane, the differences among the three in-plane magnetization curves remain relatively modest and are much smaller than the difference between the in-plane and out-of-plane magnetization. Therefore, although the magnetic easy axis may deviate slightly from the exact out-of-plane $[001]_{pc}$ axis due to the finite in-plane MA, both samples still exhibit a clearly PMA-dominated magnetism. In this sense, the easy axis remains close to the film normal rather than tilt towards the film plane. In contrast, the PAT-prepared freestanding LSMRO membrane exhibits nearly isotropic in-plane magnetic behavior. The *M-T* curves measured along the in-plane $[100]_{pc}$, $[110]_{pc}$, and $[010]_{pc}$ axes almost overlap with each other, and the corresponding *M-H* loops are also nearly indistinguishable. This result is fully consistent with the complete strain release after the PAT process.

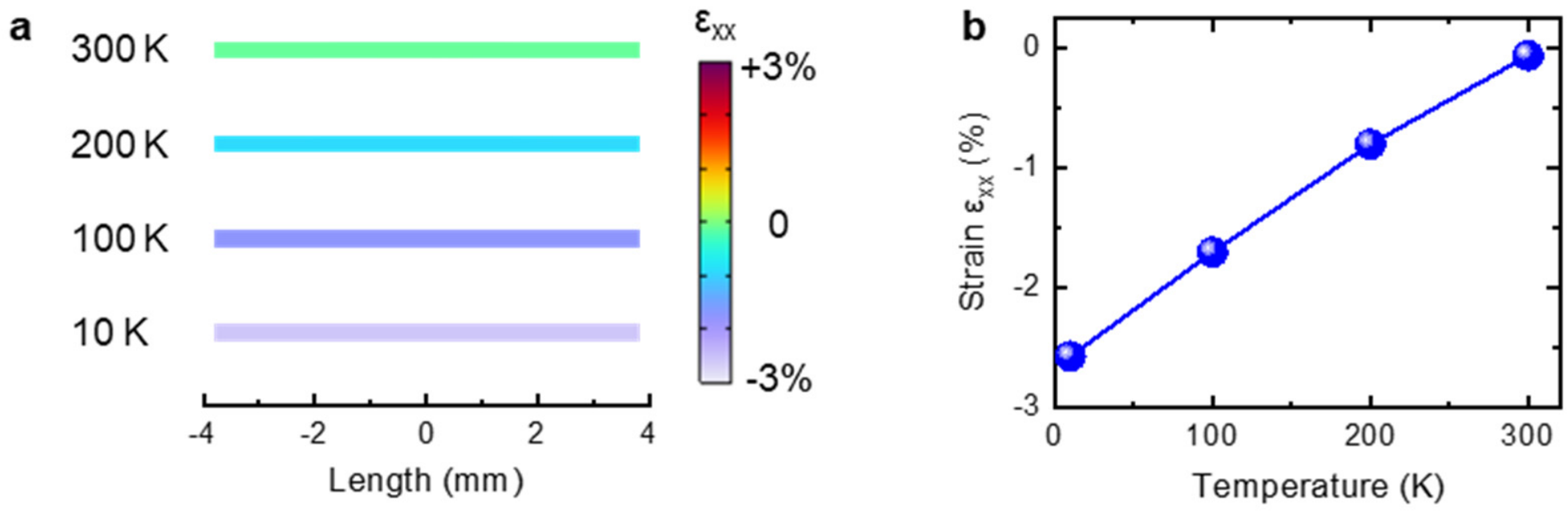

Figure S4. Finite-element simulation of thermal strain in the EAT-LSMRO membrane within a millimeter-scale LSMRO/epoxy/PET stack at various temperatures. According to the simulation, the compressive strain reaches approximately −0.8% at 200 K and further increases to about −2.57% at 10 K. This result provides a quantitative basis for our interpretation that the stronger PMA of the EAT-LSMRO membrane below 200 K originates from the additional low-temperature compressive strain generated by thermal-expansion mismatch.

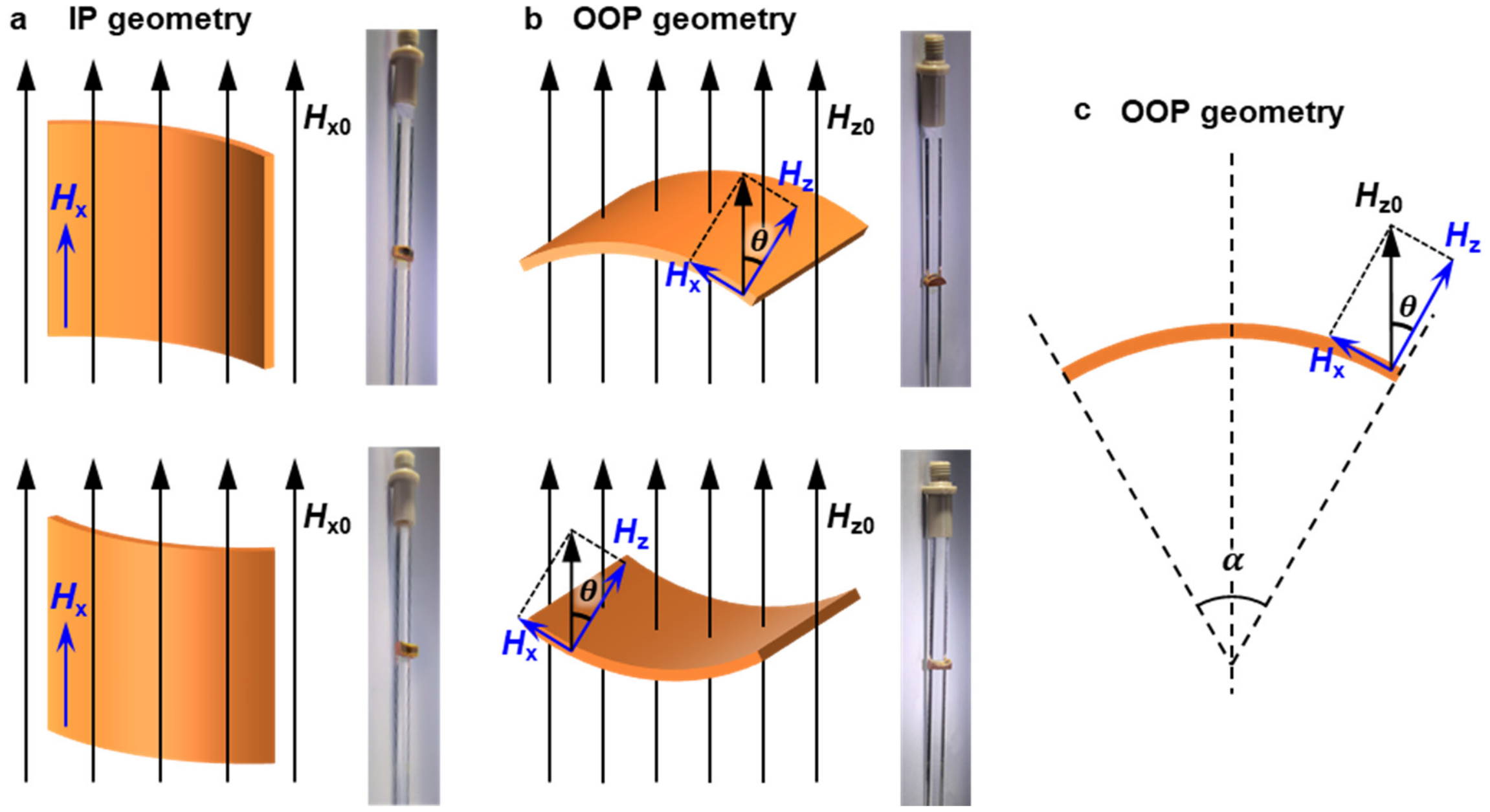

Figure S5. Schematic illustrations and photographs for the magnetic field geometries used for magnetic characterizations upon mechanical bending. (a) Nominal in-plane-field (IP) geometry (left) and physical pictures (right), in which the bending axis is parallel to the applied magnetic field H_{x0}. **(b)** Nominal out-of-plane-field (OOP) geometry (left) and physical pictures (right), in which the bending axis is perpendicular to the applied magnetic field H_{z0}. Owing to the local curvature of the membrane, the applied field is decomposed into a local out-of-plane component (H_z) and a local in-plane component (H_x), both of which vary with position along the bent arc. The upper and lower panels in (a) and (b) illustrate the convex- and concave-bending cases, respectively. **(c)** Schematic definition of the local field decomposition in the OOP geometry. At a local position with polar angle θ, the nominal field H_{z0} is resolved into the local normal component H_z and the local tangential component H_x. The total bending angle is denoted by α.

There are two distinct field geometries for magnetic characterizations during bending test. For the case of applying magnetic field in-plane (IP geometry, Figure S5a), the bending axis of the sample was aligned parallel to the magnetic field. In this configuration, the applied field H_{x0} remains parallel to the LSMRO(001) plane over the entire bent membrane. Therefore, the actual field experienced by the LSMRO membrane (H_x) is still purely in-plane ($H_x = H_{x0}$), and no additional out-of-plane component is introduced by the bending deformation. For the case of applying magnetic field out-of-plane (OOP geometry, Figure S5b), the effective out-of-plane field (H_z) applied on the bent LSMRO membrane is indeed smaller than the nominal applied field H_{z0}. Specifically, for a bent membrane with a curvature radius ***r*** and arc length ***l***, the local H_z component is given by $H_z = H_{z0}\cos\theta$, and the averaged out-of-plane component over the bent arc can be expressed as:

$$\langle H_z \rangle = \frac{1}{l}\int_{-\alpha/2}^{\alpha/2} H_{z0}\cos\theta r d\theta = H_{z0}\frac{\sin(l/2r)}{l/2r}$$

where $\alpha = l/r$ is the total bending angle. Since $\sin(x)/x < 1$ for nonzero x, the averaged out-of-plane component $\langle H_z \rangle$ is always smaller than the nominal field H_{z0}. In other words, under bending deformation, our nominal out-of-plane measurement underestimates the true tendency toward perpendicular magnetic anisotropy (PMA).

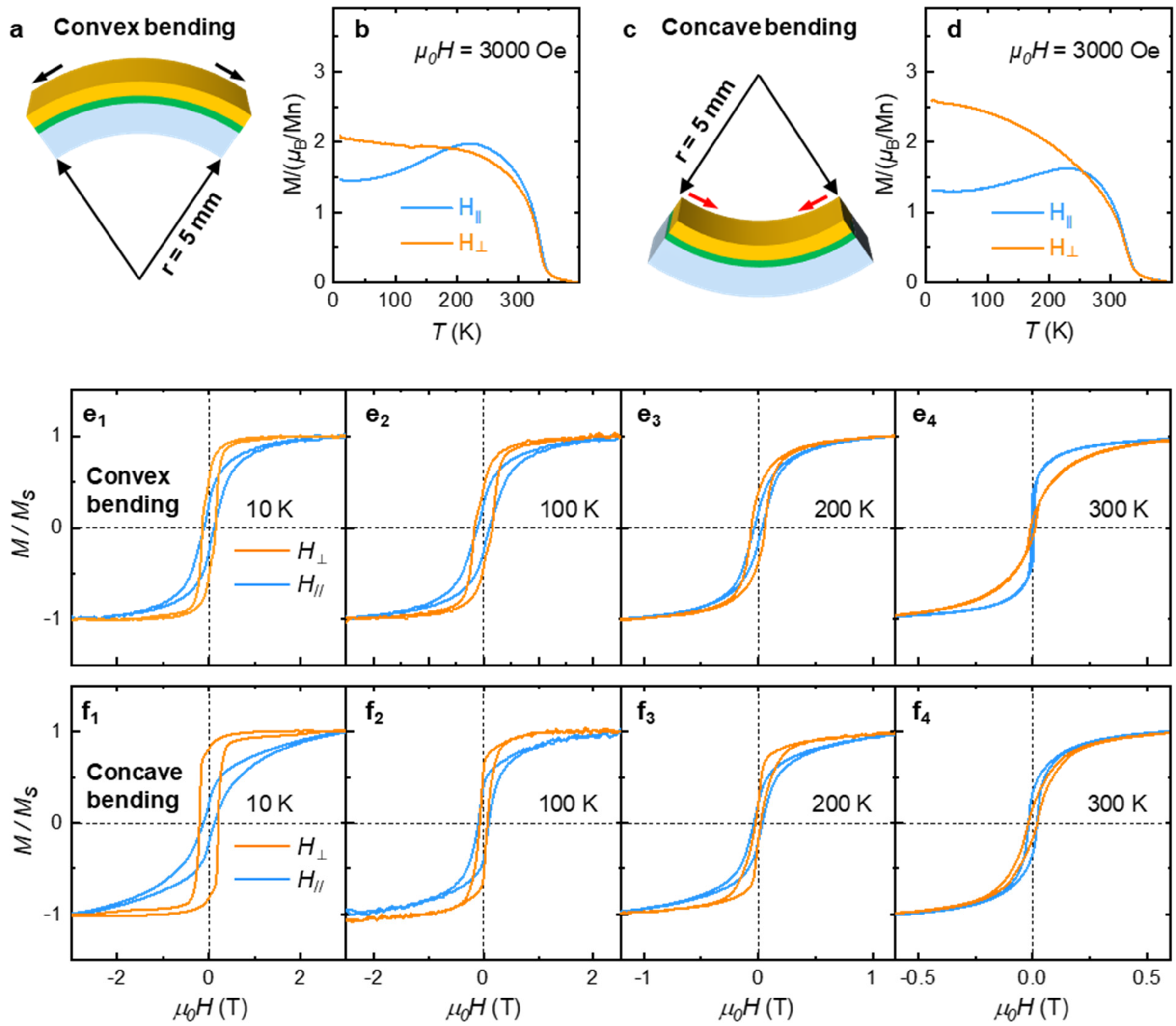

Figure S6. Magnetic characterizations of the EAT-prepared freestanding LSMRO membranes with mechanical bending. (**a,c**) Schematic illustrations of the (a) convex and (c) concave bending configurations used for introducing tensile/compressive strain states. (**b,d**) *M-T* curves measured from the EAT-LSMRO membranes under (b) convex and (d) concave bending configurations. During the *M-T* measurements, a magnetic field of 3000 Oe is applied in-plane ($H_{||}$, blue) or out-of-plane ($H_{\perp}$, orange). (**e,f**) *M-H* hysteresis loops measured at 10 to 300 K under (e) convex and (f) concave bending configuration.

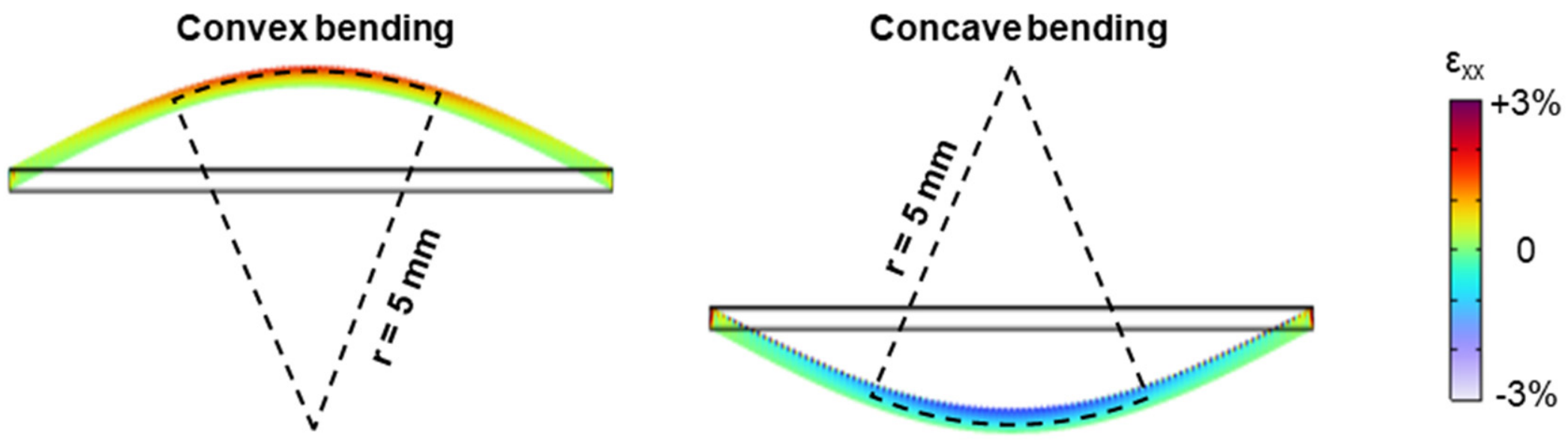

Figure S7. Finite-element simulation of the strain tensor for the bending-dependent magnetic anisotropy measurements. The model ends are constrained to suppress rigid-body motion, and the uniformly strained central region closely represents the experimental strain condition. Under convex bending, the LSMRO membrane is located on the outer side of the curved laminate and therefore experiences tensile in-plane strain. Under concave bending, the membrane is located on the inner side and therefore experiences compressive in-plane strain.

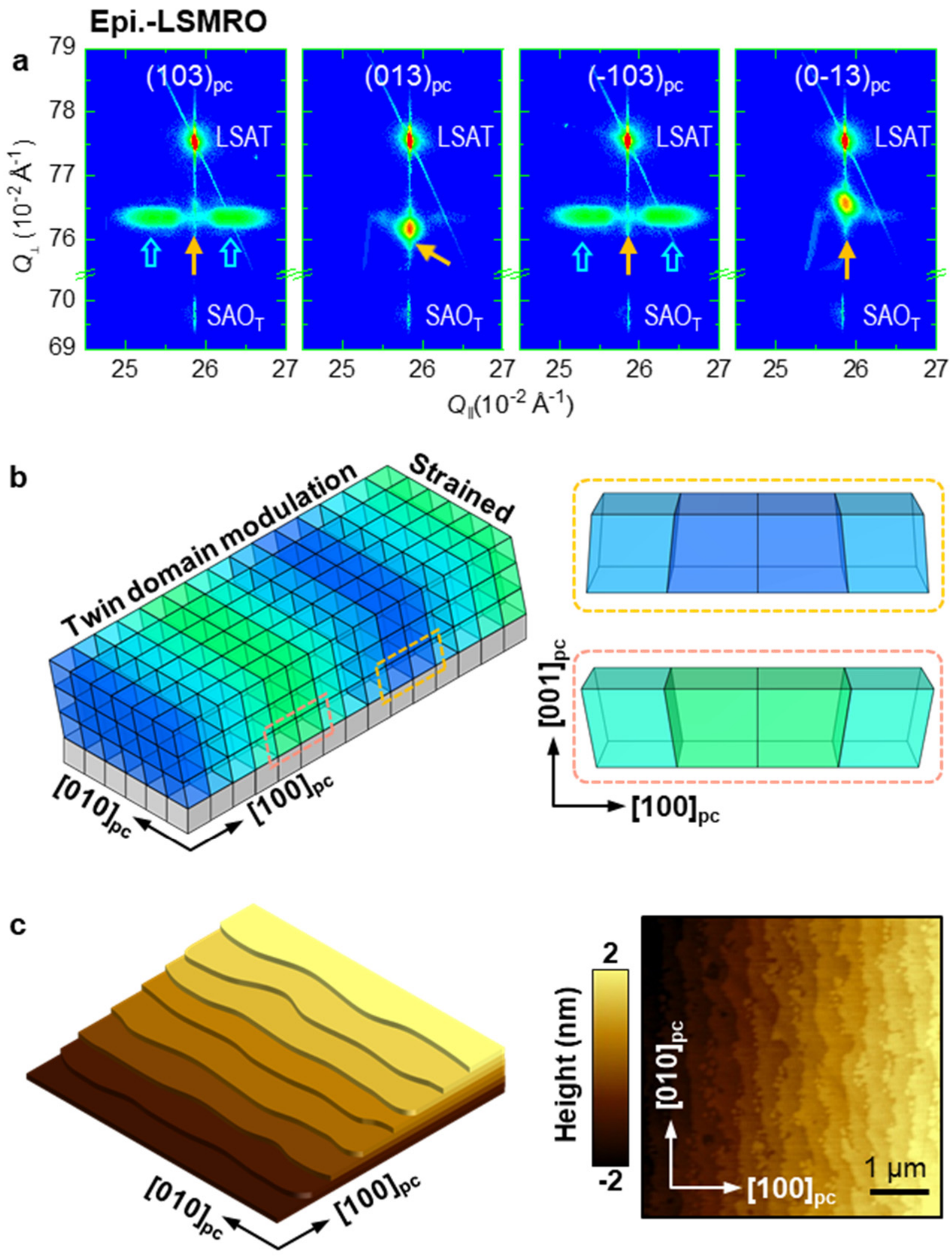

Figure S8. Formation of periodic structural modulations in a LSMRO/SAO$_T$/LSAT(001) epitaxial film (**a**) Off-specular RSMs measured from a 30 nm-thick LSMRO/SAO$_T$/LSAT(001) film around the $(013)_{pc}$, $(103)_{pc}$, $(0\text{-}13)_{pc}$, and $(\text{-}103)_{pc}$ diffractions. The LSMRO diffraction spots are marked by yellow arrows, and the satellite diffractions are marked by blue hollow arrows. Two satellite diffractions appear beside the LSMRO$(103)_{pc}$ and $(\text{-}103)_{pc}$ diffractions, while the satellites disappear around the LSMRO(0±13)pc reflections. (**b**) Schematic illustration of the structural modulation of the LSMRO/SAO$_T$/LSAT(001) film. (**c**) Schematic step terrace structure at the LSMRO thin film surface (left) and atomic force microscopy image of the LSMRO thin film surface, which clearly displays straight step-terraces along the LSMRO$[010]_{pc}$ axis.

The bulk LSMRO unit-cell has a rhombohedral symmetry. Although LSAT is cubic, the LSMRO film under compressive strain adopts a monoclinic (or distorted orthorhombic-like) structure. This monoclinic crystal structure follows the Glazer octahedral tilt system $a^+a^-c^-$, similarly to compressively strained LSMO and SRO films. This distorted structure can be understood as arising from the competition between the epitaxial clamping imposed by the substrate and the tendency of bulk rhombohedral LSMRO to recover its preferred lattice symmetry. In this situation, besides the lattice-parameter mismatch with LSAT, the rhombohedral bulk LSMRO

unit cell also has a lattice-angle mismatch with the cubic substrate, which introduces shear strain during coherent epitaxial growth. To partially release this accumulated shear strain, the film develops a periodic structural modulation. According to the position of satellite diffractions of LSMRO$(103)_{pc}$ and $(-103)_{pc}$, the periodicity of this structural modulation should be ~22 nm.[1,2]

For a film grown on an ideal cubic LSAT(001) surface, the two in-plane axes ($[100]_{pc}$ and $[010]_{pc}$) are symmetrically-equivalent. In particular, the LSAT(001) substrate has a finite miscut of ~0.1° and therefore exhibits a step-terrace surface morphology. These step edges introduce an additional uniaxial strain-relaxation tendency on the substrate surface, which lifts the equivalence between the two otherwise symmetry-equivalent modulation directions. In our sample, the step edges are oriented along one in-plane axis (we define it as $[010]_{pc}$), and thus the periodic structural modulations preferentially align with this $[010]_{pc}$ axis to minimize the elastic energy. Such step-guided alignment of structural or ferroelastic domains in perovskite oxide films has also been reported previously.[3]

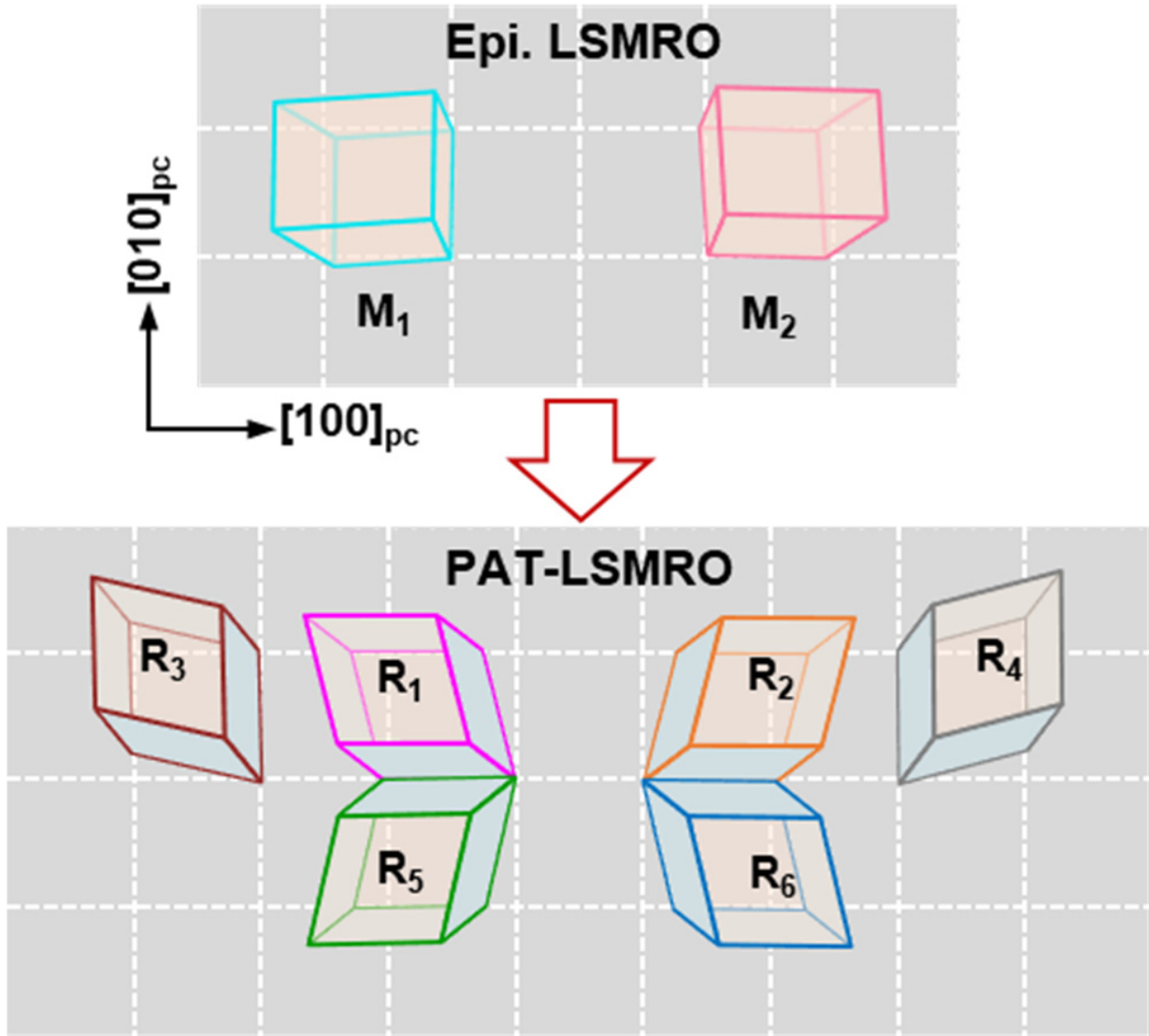

Figure S9. Schematic illustrations of the structural domain configurations in epitaxial (top) and PAT-LSMRO (bottom) membranes.

In principle, a fully relaxed R-domain structure derived from a biaxially-strained pseudocubic parent lattice can indeed exhibit up to eight domain variants. [4] However, in our case, the PAT-prepared LSMRO membrane does not evolve from such a biaxially-strained pseudocubic parent lattice. Before the water release procedure, the epitaxial LSMRO/SAO_T/LSAT(001) film is fully strained along the $[010]_{pc}$ axis but already adopts a uniaxial shear-strain relaxation and a periodic twin-domain modulation along $[100]_{pc}$ axis. Therefore, the R-domains appearing after full strain release must evolve from this pre-existing twin-domain precursor state. As illustrated in Figure S9, the two precursor monoclinic twin domains, denoted as Domain M_1 and M_2, first evolve naturally into four R-domain variants, R_1–R_4. These four R-domain variants are all tilted towards the $[010]_{pc}$ axis, which is compatible with the tilting direction of Domains M_1 and M_2 prior to water release. After the PAT process fully relaxes the clamped strain along $[010]_{pc}$ axis, an additional lattice flipping towards the $[0\bar{1}0]_{pc}$ direction (opposite tilt around [100]pc axis) may become energetically accessible, giving rise to two more R-domain variants, R_5 and R_6. In contrast, the remaining two *R*-domain variants (R_7 and R_8) are expected to evolve from a $[100]_{pc}$-clamped precursor domain configuration, which is inconsistent with the twin-domain modulations in our epitaxial LSMRO/SAO_T/LSAT(001) film (fully-strained along $[010]_{pc}$ axis). The formation of R_7 and R_8 would therefore involve a substantially higher elastic-energy cost. We thus believe that these two variants are strongly suppressed, which explains why only six rhombohedral domains are observed experimentally.

	Lattice Constant (Å)		Lattice mismatch (%)
	**Film**	**Substrate**	
	**$La_{2/3}Ca_{1/3}MnO_3$**	**NGO(001)**	
a_O	5.472	5.433	-0.71
b_O	5.457	5.503	+0.85
	**$SrRuO_3$**	**LSAT(001)**	
a_{pc}	3.93	3.868	-1.58
b_{pc}	3.93	3.868	-1.58

Table S1. Lattice constants of LCMO and SRO oxides, and the lattice mismatches between these oxides and NGO(001) or LSAT(001) substrates.

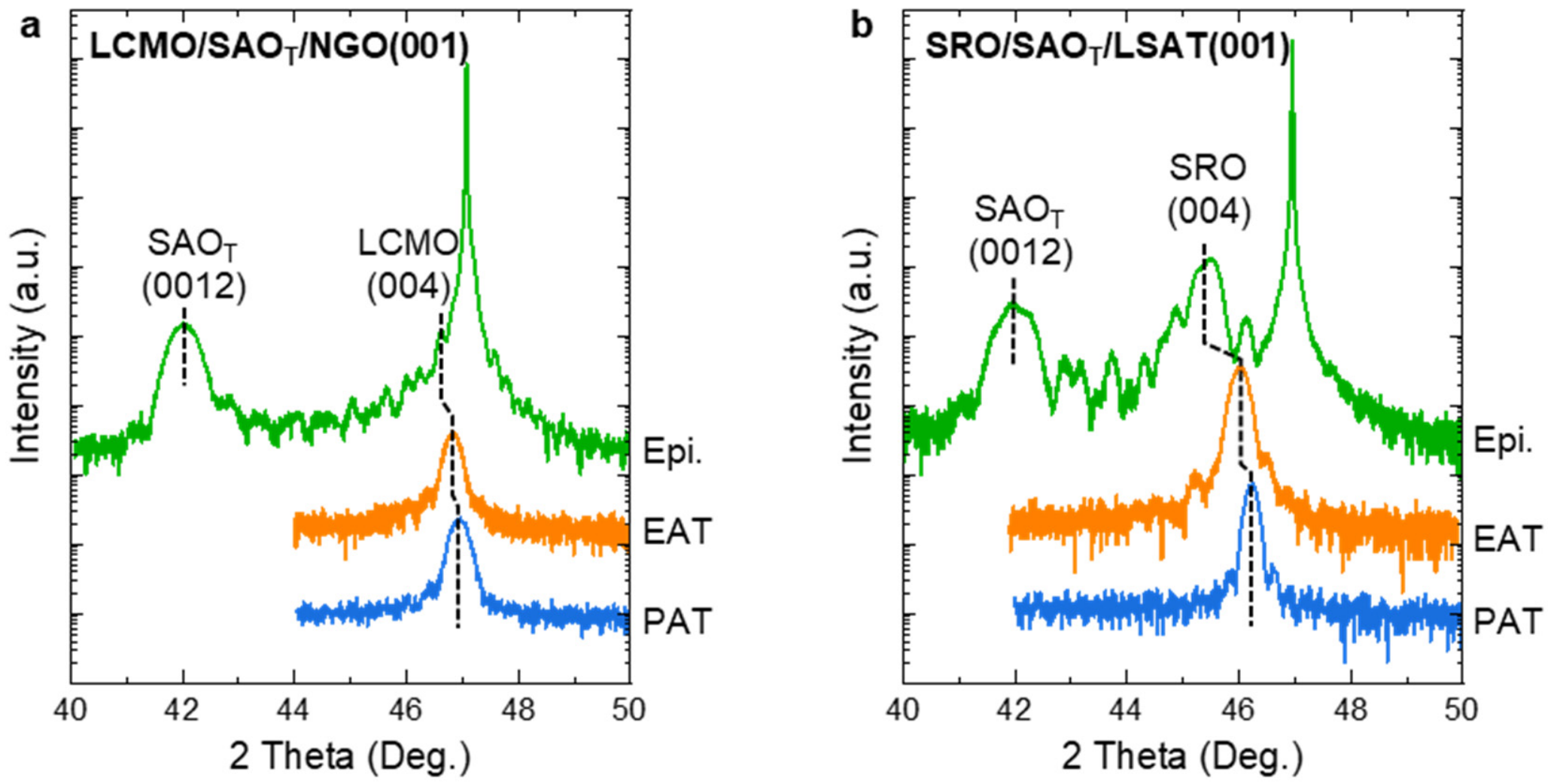

Figure S10. XRD 2θ-ω linear scans of the as-grown and freestanding (**a**) LCMO and (**b**) SRO films. Here, the (002) diffraction peaks of all freestanding films shift toward higher Bragg angles, and the PAT-prepared membranes shows more pronounced peak shifts towards higher Bragg angle, indicating that the out-of-plane lattice constant tend to restore to the bulk values.

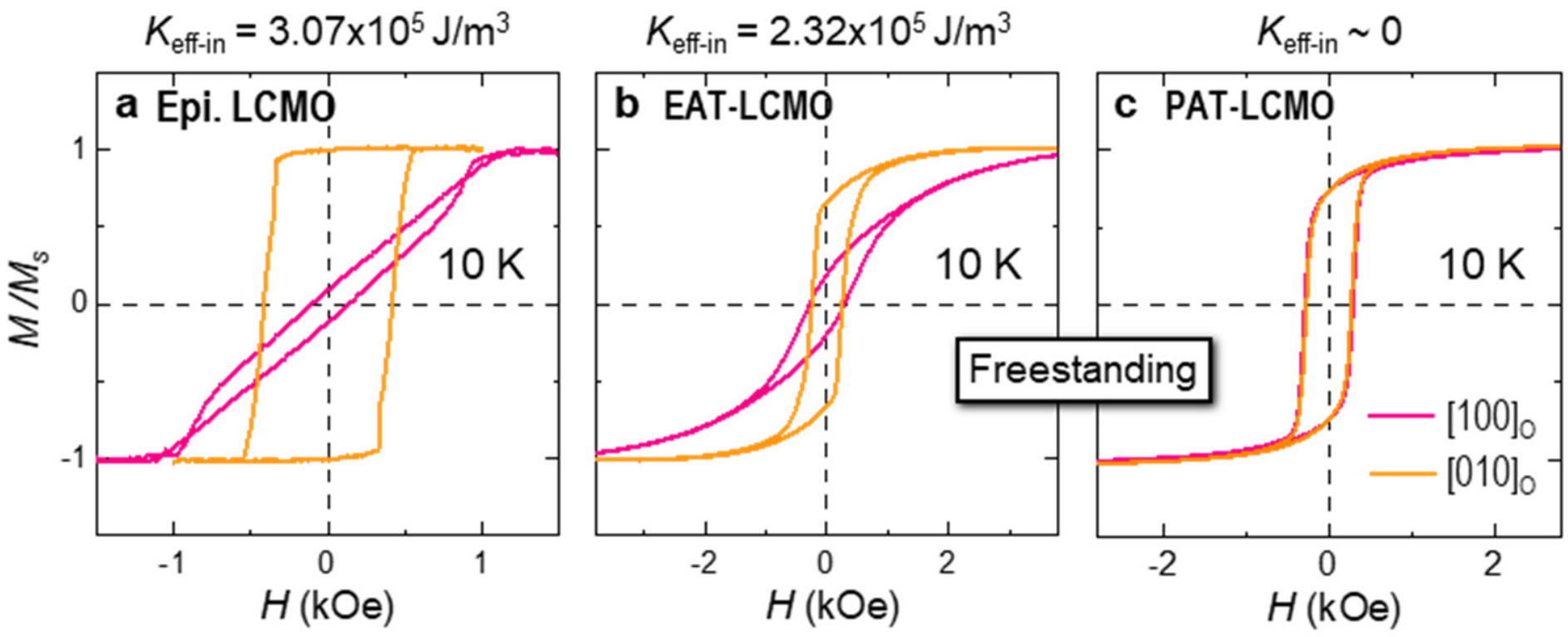

Figure S11. (**a-c**) *M-H* loops measured at 10 K from (a) an as-grown LCMO/SAO_T/$NGO(001)_O$ epitaxial film, (b) an EAT-prepared freestanding LCMO membrane, and (c) a PAT-prepared LCMO membrane. During the measurements, we applied *H* along the in-plane $[100]_O$ and $[010]_O$ axes. The effective in-plane magnetic anisotropy constants ($K_{eff\text{-}in}$) values for these three samples are also labeled.

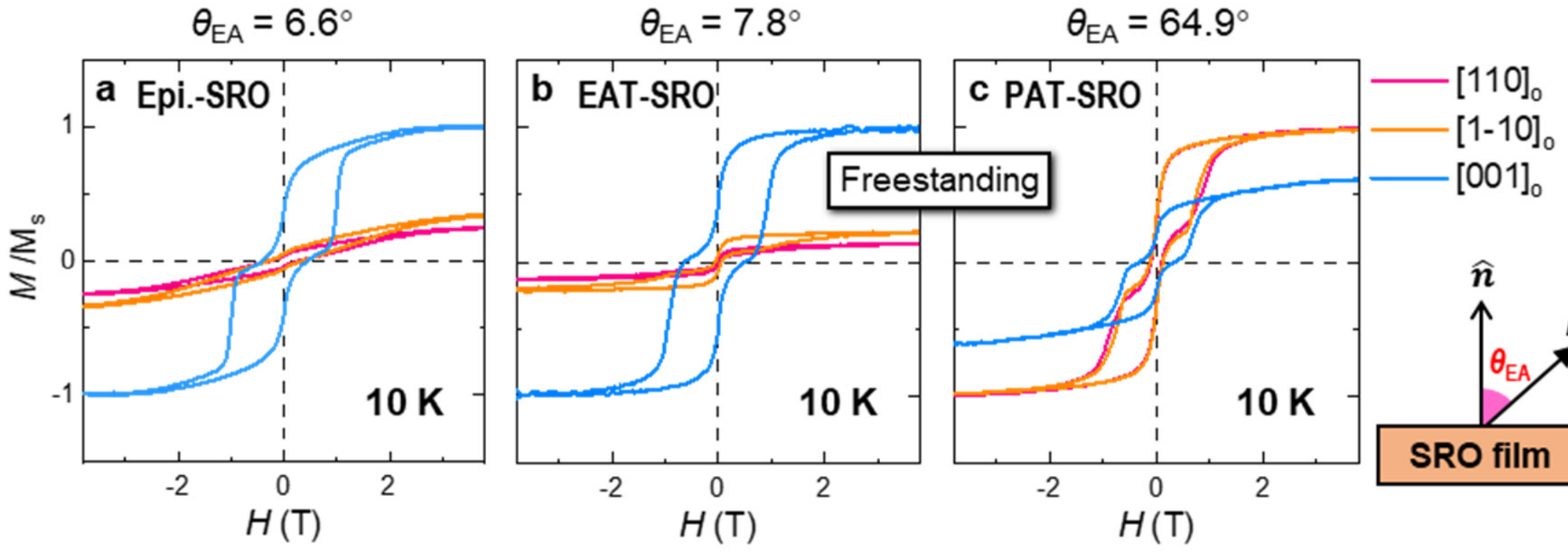

Figure S12. (**a-c**) *M-H* loops measured at 10 K from (a) an as-grown SRO/SAO_T/LSAT$(001)_O$ epitaxial film, (b) an EAT-prepared freestanding SRO membrane, and (c) a PAT-prepared SRO membrane. During the measurements, we applied *H* along three orthogonal axes: SRO$[110]_O$, SRO$[1\text{-}10]_O$, and SRO$[001]_O$ axes, which correspond to the LSAT$[100]_{pc}$, LSAT$[010]_{pc}$, and LSAT$[001]_{pc}$ axes, respectively.

The strong magnetocrystalline anisotropy of SRO results in a high saturation field (H_S) higher than 10 T, which is beyond the maximum magnetic field we can apply (~7 T). Therefore, we cannot precisely determine the K_{eff} values for the SRO films/membranes. Instead, we determine the angle between the film normal ($\hat{n}$) and magnetic easy axis (defined as θ_{EA}) by extracting the *M* values at *H* = 3000 Oe along the three orthogonal axes (denoted as $M_{[001]o}$, $M_{[110]o}$, and $M_{[1\text{-}10]o}$).[5] Specifically, the θ_{EA} is calculated by the following equation:

$$\theta_{EA} = \arctan\frac{\sqrt{(M_{[110]o})^2 + (M_{[1-10]o})^2}}{M_{[001]o}}$$

The calculated θ_{EA} values are labeled in (a-c). Note that the epitaxial SRO film and EAT-prepared SRO membrane exhibit rather small $\theta_{EA} < 8^o$, signifying a strong PMA. In sharp contrast, the PAT-prepared SRO membrane exhibits a large θ_{EA} up to 64.9^o, which can be attributed to the intrinsic magnetocrystalline anisotropy of bulk SRO. Note that the tilted PMA in EAT-prepared SRO and LSMRO can be ultilized for fabricating spin-orbital torque device with field-free switcing behavior (see schematic illustrations in **Figure S13**).[6]

Notably, the *M-H* curves for all the SRO samples display a double-loop feature. The slim component can be attributed to oxygen-deficient regions near the SRO/SAO_T heterointerfaces, formed due to interfacial diffusion of oxygen vacancies during the pulsed laser deposition.[7]

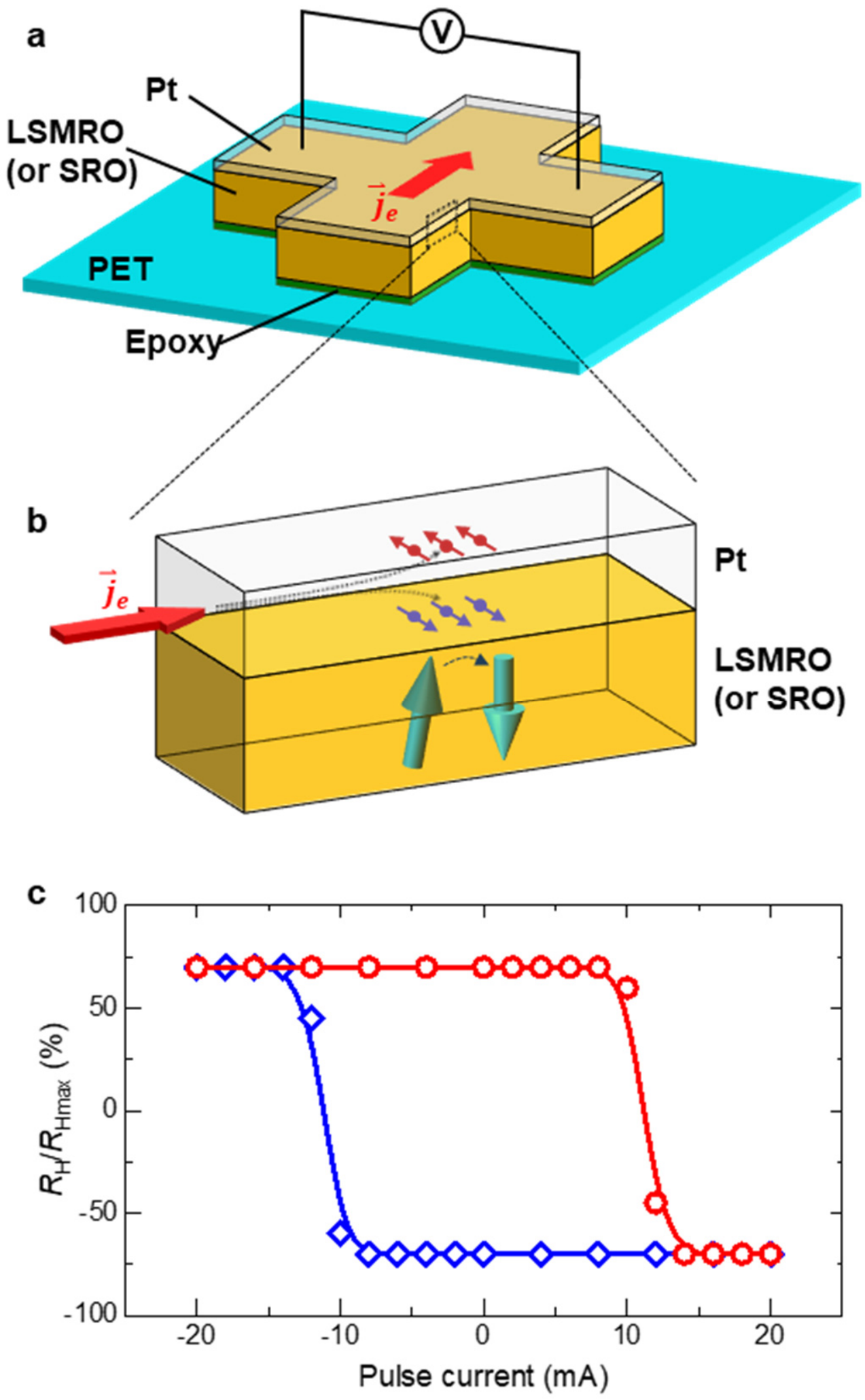

Figure S13. (**a,b**) Schematic illustration of a Hall bar fabricated based on EAT-prepared magnetic membrane (LSMRO or SRO)/Pt bilayer heterostructure. (**b**) Schematic illustration SOT-induced field-free switching of EAT-prepared LSMRO/SRO with tilted magnetic moment. (**c**) Schematic anomalous Hall resistivity (R_H) vesus pulse current curve corresponding to the SOT-induced field-free switching. The R_H is normalized by the maximum swiching amplitude of R_H (R_{Hmax}).

References

1. D. Lan, P. Chen, C. Liu, et al. "Interfacial control of domain structure and magnetic anisotropy in $La_{0.67}Sr_{0.33}MnO_3$ manganite heterostructures," *Physical Review B* 104 (2021): 125423.

2. A. Vailionis, H. Boschker, W. Siemons, et al. "Misfit strain accommodation in epitaxial ABO_3 perovskites: Lattice rotations and lattice modulations," *Physical Review B* 83 (2011): 064101.

3. H. Boschker, M. Mathews, P. Brinks, et al. "Uniaxial contribution to the magnetic anisotropy of $La_{0.67}Sr_{0.33}MnO_3$ thin films induced by orthorhombic crystal structure." *Journal of Magnetism and Magnetic Materials* 323 (2011): 2632-2638

4. Y. Tao, E. Peng, Q. He, et al. "Simulation of three dimensional diffraction patterns as aid of structural analysis for complex epitaxial films," *Scientific Reports* 15 (2025): 18328.

5. H. Peng, N. Lu, S. Yang, et al. "A Generic Sacrificial Layer for Wide-Range Freestanding Oxides with Modulated Magnetic Anisotropy," *Advanced Function Materials* 32 (2022): 2111907.

6. Y. Jo, Y. Kim, S. Kim, et al. "Field-free spin-orbit torque magnetization switching in a single-phase ferromagnetic and spin Hall oxide" Nano Letters 24 (2024): 7100–7107.

7. J. Lu, L. Si, Q. Zhang, et al. "Defect-Engineered Dzyaloshinskii-Moriya Interaction and Electric-Field-Switchable Topological Spin Texture in $SrRuO_3$," *Advanced Materials* 33 (2021): 2102525.
